# The larval gut as a mirror: bacterial community composition and functional potential of mayfly larvae reflect site and seasonality differences

**DOI:** 10.1093/ismeco/ycag192

**Published:** 2026-07-03

**Authors:** Rubén Martínez-Cuesta, Rebecca Hoess, Juergen Geist, Michael Schloter, Stefanie Schulz

**Affiliations:** Chair of Environmental Microbiology, TUM School of Life Sciences, Technical University of Munich, Emil-Ramann-Straße 2, 85354 Freising, Bavaria, Germany; Research Unit Comparative Microbiome Analysis, Helmholtz Zentrum Munich, Ingolstädter Landstraße 1, 85764 Neuherberg, Bavaria, Germany; Aquatic Systems Biology Unit, Technical University of Munich, Mühlenweg 22, 85354 Freising, Bavaria, Germany; Aquatic Systems Biology Unit, Technical University of Munich, Mühlenweg 22, 85354 Freising, Bavaria, Germany; Chair of Environmental Microbiology, TUM School of Life Sciences, Technical University of Munich, Emil-Ramann-Straße 2, 85354 Freising, Bavaria, Germany; Research Unit Comparative Microbiome Analysis, Helmholtz Zentrum Munich, Ingolstädter Landstraße 1, 85764 Neuherberg, Bavaria, Germany; Research Unit Comparative Microbiome Analysis, Helmholtz Zentrum Munich, Ingolstädter Landstraße 1, 85764 Neuherberg, Bavaria, Germany

**Keywords:** freshwater trophic chains, land use, long-read metagenomic sequencing, host microbiome, filter-feeding invertebrates

## Abstract

Land use intensification is a major driver of biodiversity loss across ecosystems, yet its consequences for host-associated microbiomes in freshwater food webs remain poorly understood. In this case study, we used the gut microbiome of mayfly larvae (*Ephemera danica*) as a sensitive biological interface to assess how site-specific adjacent land use types shape microbial community composition and functions in stream ecosystems. Larvae were sampled in summer and autumn from sites adjacent to forest, extensive grassland, and intensive agriculture along the Otterbach stream (Bavarian Forest, Germany). Combining 16S ribosomal RNA (rRNA) amplicon sequencing with long-read metagenomics, we show that site-specific land use, in interaction with seasonality, significantly restructures larval gut bacterial communities without affecting alpha diversity. Rather than introducing distinct agriculturally derived taxa, agricultural land use acted as a selective environmental filter, enriching bacterial groups with specific functional traits. Taxa enriched in the sites adjacent to agricultural sites harboured genes involved in complex carbon and xenobiotic degradation, short-chain fatty acid production, efflux pumps, and stress response. These functional signatures were further supported by 14 metagenome-assembled genomes linked to these enriched taxa. Together, our results reveal that site in combination with seasonality not only reshaped bacterial community composition without affecting alpha diversity but also triggered shifts in the abundance of genes involved in microbial-host interactions and degradation pathways in *E. danica* larvae. This study also highlights the larval gut microbiome as a sensitive indicator of environmental change, suggesting that environmental microbial shifts may have cascading consequences for freshwater trophic interactions and ecosystem functioning.

## Graphical abstract

**Graphical Abstract:**
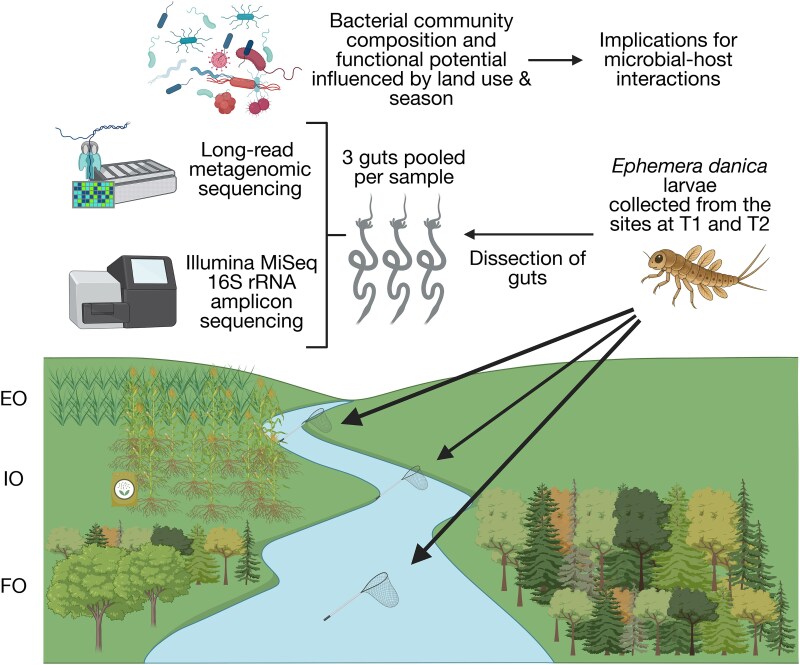

Land use change and intensification are major drivers of biodiversity loss and ecosystem functioning both in terrestrial and adjacent aquatic ecosystems [1, 2]. Agricultural activities increase the input of nutrients, organic matter, and pollutants into nearby streams, reducing water quality [3] and modifying the structure and functioning of aquatic communities [4]. Such changes can propagate across trophic levels, including aquatic insects, which are often used as indicators of freshwater ecosystems’ health [5, 6]. Species with a collector-filtrator feeding strategy may be especially exposed to adjacent land use, as they continuously process suspended organic matter and microorganisms from the water column [7]. These inputs may influence the composition and function of the gut microbiome, a key component of host physiology, immune regulation, and protection against pathogens [8].

To close this gap of knowledge, we focused on the larval stage of *Ephemera danica*, a mayfly whose larvae are widespread across European streams [9]. Given previous studies demonstrating that environmental conditions can shape aquatic host-associated microbiota, such as in *Daphnia* and corals [10, 11], we hypothesized that the gut microbiome of mayfly larvae reflects differences in adjacent land use. Specifically, we expect stream sites influenced by intensive agriculture, extensive grasslands, or forests to harbour distinct microbial community compositions and functional profiles driven by differences in nutrient runoff, availability of labile carbon sources, and micropollutants, which may impose selective pressures on gut microbial assemblages [12], in which the enriched microbial taxa will harbour functions related to degradation pathways and host–microbe interactions [13]. We deployed 16S rRNA amplicon sequencing and long-read metagenomics to characterize the bacterial communities of dissected mayfly larvae gut samples, which were collected from stream sites adjacent to different land use types at two sampling times (Fig. S1).

A total of 614 034 16S rRNA amplicon sequences were retained after processing and contaminants removal (Table S1). Larval gut bacterial α-diversity (Hill numbers) yielded no significant differences across sites, sampling times, and their interaction (Fig. S2). However, the interaction of land use and sampling time significantly affected (PERMANOVA, *P* = .001) community composition (Fig. 1A). This interaction likely reflects the seasonal dynamics of the agricultural activity in the area, such as fertilization events taking place in spring and early summer, and a cease in fertilization and the harvest of crops in autumn. In addition, higher rainfall in summer compared to autumn (Fig. S3) likely amplified the leaching of agricultural runoff into the stream, verified by higher stream nutrient concentrations at the agriculturally influenced sites [14]. This observation aligns with previous research [15], in which seasonality had a greater impact on water quality than land use in watersheds. However, given the limitation of one site per land use, other factors could have also explained the differences, such as thermal buffering due to vegetation cover or differences in litter input. Sampling time also had a significant effect on β-diversity, which indicates that larval gut bacterial communities underwent a temporal turnover independent of the site effect. Beta dispersion was higher in summer and increased along the stream (Fig. S4). Nevertheless, differences between sites were not significant at any sampling time.

**Figure 1:**
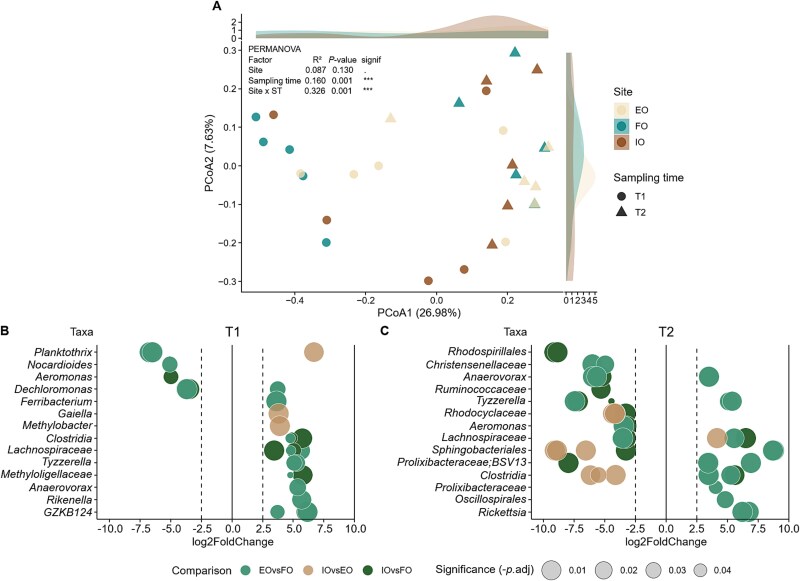
Influence of adjacent land use and sampling time on the gut bacterial communities of *E. danica* (5 replicates × 3 sites × 2 sampling times, *N* = 30). A β-diversity based on the bray-Curtis distance with the significance of study variables on dissimilarity assessed by PERMANOVA. Colours indicate the adjacent sites under different land use types: EO (extensive grassland), FO (forest) and intensive farming (IO), which were located 3.5-4 km apart. Shapes represent sampling times, summer (T1) and autumn (T2). Differentially abundant ASVs (as bubbles) labelled according to the lowest possible taxonomic rank in summer (T1; B) and autumn (T2; C). Values are displayed as log2 fold change, in which a positive value indicates higher abundance in the first listed site of each pair. Dashed lines represent the ±2.5 log2 fold change significance threshold. Colours correspond to the pairwise comparison of the sites (EO vs. FO, IO vs. EO, IO vs. FO). Bubble size is inversely proportional to the adjusted *P* value of each differentially abundant ASV.

Differential abundance analyses identified specific taxonomic shifts (Fig. 1B, C). In summer, only 6 enriched ASVs were found at the forest in the summer sampling time, when comparing its samples against those from the intensive and extensive sites (Fig. 1B). These belong to *Planktothrix, Nocardioides, Aeromonas*, and *Dechloromonas*. Conversely, more ASVs were enriched in the samples from the intensive and extensive sites when compared against those from the forest (Fig. 1B). These ASVs were affiliated with members of *Clostridia, Lachnospiraceae, Tyzzerella, Anaerovorax*, and *Rikenella*. By autumn, these strong shifts at the intensive and extensive sites were no longer evident. Instead, more ASVs were enriched at the forest site, some overlapping with the extensive site, including *Rhodospirillales, Rhodocyclaceae, Anaerovorax*, or *Prolixibacteraceae. Clostridia* remained particularly enriched at the extensive site compared to both the forest and intensive sites.

Consistent with previous studies showing how aquatic host microbiomes adapt to environmental modifications [16–18], our results from the Otterbach case study also demonstrate pronounced shifts in gut bacterial community composition. However, enriched taxa were not directly traceable to agricultural sources. This suggests that agricultural inputs, as detected in the stream physicochemical properties at each site [14], primarily act as a selective environmental filter, favouring functionally adapted microbial groups rather than simply introducing foreign taxa.

Metagenomic sequencing resulted in 4 012 653 annotated open reading frames after the functional annotation of the processed reads (Table S2). Among the targeted functional groups (Table S3), we identified 33 genes related to degradation and microbial-host interaction harboured by the bacterial taxa highlighted in the previous differential abundance analyses (Fig. 1B, C). Of those, we detected genes involved in complex carbon and xenobiotics degradation (*glcD, GST, EG, xynA, xynB, abfA, pcaC, pcaB, pccB, bglX, phnJ*, and *boxC*), efflux pumps (*mexA, mexB, tolC, oprM*), short-chain fatty acids (SCFAs) production (*ackA, atoA, atoD, buk, pct, pduC, pduE, por, pta, ptb, tesA*) and stress response (*ackA, atoA, atoD, buk, pct, pduC, pduE, por, pta, ptb, tesA*) (Fig. 2A). Overall, the abundance of genes associated with degradation and host–microbe interaction functions was consistently higher at the intensive site at both sampling times (Fig. S5; Tables S4 and S5). Except efflux pumps genes, which showed a higher abundance at the extensive site in summer, but only significant when compared to the samples from the forest site (Tables S4 and S5).

**Figure 2:**
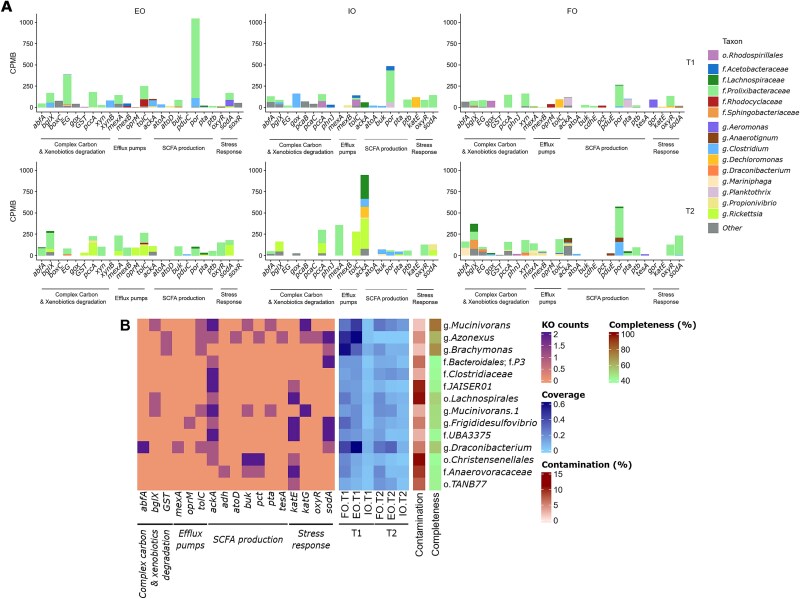
Distribution of degradative and host–microbe interaction genes and characterization of recovered MAGs. **A** abundances (coverage per million bases) of genes within functional groups associated with degradative and host–microbe interactions. Colours indicate the taxonomic origin of the detected genes. **B** Heatmap summarizing properties of MAGs (≥40% completeness, ≤10% contamination) recovered from the larval gut metagenomes, showing completeness, contamination, mean coverage across sites and sampling times, and KEGG Orthology (KO) counts of the genes mentioned in **A**.

Linking taxonomic shifts to functional potential (Fig. 2A), the identified taxa differed markedly in the functions they harboured. *Prolixibacteraceae* were consistently detected across sites and sampling times and carried genes from all functional categories, including *EG* (complex carbon and xenobiotics degradation), *mexA* (efflux pumps), *sodA* (stress response), and *por* (SCFA production), indicating a high degree of functional versatility within the larval gut microbiome. In contrast, other taxa enriched under specific adjacent land use conditions exhibited more specialized functional profiles, such as *Lachnospiraceae* and *Rhodocyclaceae*, which were primarily associated with SCFA production and efflux pump genes, respectively, consistent with their previously reported roles in *Ephemeroptera* gut microbiota [19].

Beyond these dominant and specialized taxa, reads assigned to *Rickettsia* were associated with genes involved in fatty acid metabolism (*ackA, pccA*) and stress-related functions (*GST, tolC, oprM, sodA*), pointing to a potential role in host energy metabolism and stress tolerance. This aligns with previous reports describing beneficial effects of *Rickettsia* in insects, including contributions to nutrition and defence [20], despite its common classification as an intracellular parasite. Notably, such function-associated *Rickettsia* reads were absent from samples collected at the forest site.

Furthermore, we retrieved 14 metagenome-assembled genomes (MAGs) from the metagenomes that met previously defined quality thresholds [21]. Several MAGs belonged to taxonomic groups also identified among ASVs enriched under adjacent land use. In particular, MAGs classified as *Azonexus* (family *Rhodocyclaceae*), *Draconibacterium* (family *Prolixibacteraceae*), the order *TANB77* (class *Clostridia*), and the family *JAISER01* (order *Lachnospirales*) corresponded to taxa previously detected in the differential abundance analyses and associated with genes involved in degradation processes and microbial-host interactions. Members of *Rhodocyclaceae*, including *Azonexus* and *Brachymonas*, are metabolically versatile freshwater bacteria often involved in nitrogen cycling and organic compound degradation [22, 23]. In contrast, *Prolixibacteraceae*, including *Draconibacterium* and *Mucinivorans*, are known for their capacity to fix nitrogen [24], degrade complex polysaccharides [25], suggesting a role in the breakdown of organic substrates ingested by the larvae. MAGs affiliated with *Clostridia* (order *TANB77*) and *Lachnospirales* (family *JAISER01*) further indicate the presence of fermentative bacteria capable of producing short-chain fatty acids, as shown in other insects [26, 27]. Genomic neighbourhood analyses (Fig. S6) revealed genes related to nutrient acquisition (*araA, araD, susC, susD, GBA, ntpI*), nutrient biosynthesis (*folB, cobH–cbiC, argH, cysH, OTC*), and stress protection (*dps, mntP, nhaC, clpP, hslR, tig*), which indicate that members of the larval gut microbiome possess metabolic capabilities for substrate utilization, nutrient transformation, and tolerance to environmental stress within the host digestive tract.

This study showcases how complex interactions between site-specific adjacent land use and seasonal dynamics acted as a selective filter, restructuring the gut bacterial community composition of *E. danica* while maintaining a stable alpha diversity. Enriched taxa carried genes involved in complex carbon and xenobiotic degradation, short-chain fatty acid production, efflux pumps, and oxidative stress responses. Future research should link these microbiome changes to host fitness and resilience, as in previous work on other aquatic invertebrates [28]. Moreover, this study can guide further experimental work on large-scale replicated multi-stream studies to further separate land-use effects from other site-specific drivers and/or controlled small-scale mesocosm experiments to improve our mechanistic understanding, respectively.

## Supplementary Material

20260630_supp_mat_larvae_brief_comm_ycag192

## Data Availability

The bioinformatic scripts used in the experiment can be found in GitHub (https://github.com/rubmc97/larvae_exp_BLIKII). The raw reads from the 16S rRNA amplicon and long-read metagenomic sequencing can be found in the NCBI’s sequence read archive repository under the BioProject IDs PRJNA1424985 and PRJNA1424986, respectively.
