# Supplementary material for: The larval gut as a mirror: bacterial community composition and functional potential of mayfly larvae reflect site and seasonality differences
Source: ISME Commun. 2026 Jul 3;6(1):ycag192. doi: 10.1093/ismeco/ycag192 (PMC13455633; doi:10.1093/ismeco/ycag192)
Supplement: Supplementary file 1 [file 20260630_supp_mat_larvae_brief_comm_ycag192.docx]

**The larval gut as a mirror: bacterial community composition and functional potential of mayfly larvae reflect site and seasonality differences**

Rubén Martínez-Cuesta^12*^; Rebecca Hoess^3^; Juergen Geist^3^; Michael Schloter^12^; Stefanie Schulz^2^

**Materials and methods**

**Experimental site and sample collection**

*E. danica* larvae from the same larval stage were collected using a dip net and the standard kick sampling technique at two sampling points in 2023 (August and November) from three sites along the Otterbach stream in the Falkensteiner Vorwald area of the Bavarian Forest (Germany). The larvae were transported in river water from the same sites and stored at 4 °C until dissection on the following day. The Otterbach stream sites included the sites which were previously described in Martínez-Cuesta et al. (2025): (i) a forest site mainly composed of *Picea abies* and *Sambucus nigra* (FO; 49°03'45" N, 12°16'29" E – 378 m), (ii) an extensive grassland fertilized twice and mowed once per year (EO; 49º06’09” N, 12º21’30” E – 473 m), and (iii) an intensive conventional agricultural site with corn (*Zea mays*) plantations (IO; 49°04'51" N, 12°18'52" E – 443 m). In addition, the abiotic and physiochemical properties of the sites were also included in this previous study [1], in which the effect of the adjacent agricultural land use was evidenced by higher electric conductivity (EC) due to the runoff of dissolved fertilizer salts and soil minerals.

**Larval guts dissection**

The larvae were washed with sterile phosphate-buffered saline (PBS) and 70% ethanol before being placed on sterile petri dishes for dissection following a previously established protocol [2]. Briefly, sterile Dumont forceps were used to fix the larvae and extract the guts under a stereomicroscope at a magnification of 2x. The dissected guts were placed in extraction tubes and frozen at -80°C until the DNA extraction. Three larvae guts were pooled to constitute each replicate given DNA yields of trial samples and previous literature on insects [3,4]. Negative controls were taken from PBS vessels placed around the extraction spot. In total, 90 larvae were dissected

$(3 pooled guts per sample \times5 samples per site \times3 sampling sites \times2 sampling times)$.

**DNA extraction and library preparation**

Total genomic DNA (gDNA) was extracted from the dissected larval guts using the NucleoSpin® Soil Kit (Macherey-Nagel, Düren, Germany) following the manufacturer’s guidelines. The SL1 buffer was used for the chemical cell lysis, and the Precellys24 homogenizer (Bertin Technologies, Montigny-le-Bretonneux, France) was used for the mechanical cell lysis. Bead beating tubes with kit reagents and without sample were used as negative extraction controls. 30 μL of diethyl pyrocarbonate (DEPC) water was used to elute the extracted DNA. The bead-beating step was reduced to 30 s to avoid the degradation of long-fragments. DNA sample concentrations were measured using the Qubit 1x dsDNA HS assay kit (Thermo Fisher Scientific, Bremen, Germany). DNA quality and size were checked with a NanoDrop ® ND-1000 system (Thermo Fisher Scientific, Bremen, Germany) and electrophoreses in 1.5% (w/v) agarose gels. Furthermore, DNA fragment lengths were examined with a 5200 Fragment Analyzer System (Agilent Technologies, Santa Clara, CA, USA).

**16S rRNA amplicon sequencing, processing and analyses**

For the examination of the larvae gut bacterial communities and their diversity, we followed the quality guidelines of the 16S rRNA Metagenomic Sequencing Library Preparation protocol (Illumina, San Diego, CA, USA) and the previously described steps and specifications [5]. Briefly, 5 ng of DNA extracts were amplified using the primer pair for bacteria 008F (5′-AGAGTTTGATCCTGGCTCAG-3′) and 343R (5’-CTGCTGCCTCCCGTAG-3’), which target the V1-V2 [6] region of the 16S rRNA gene. Previous studies have deployed this primer pair to assess host bacterial communities [7]. Moreover, preliminary tests indicated a reduced host off-target amplification when compared to the typically used 515F/806R primer set (data not shown).

The PCR amplification was performed using 5 ng of template DNA. Each PCR consisted of 25 µL which contained 12.5 µL of NEB Next High-Fidelity Master Mix (Thermo Fisher Scientific, Bremen, Germany), 0.5 µL of each primer at 10 pmol/µl, 2.5 µL of 3% BSA, 1 µl of the template DNA and 8 µL of DEPC water. The PCR thermal profile was as follows: 98 °C for 1 min, followed by 25 cycles of 98 °C for 10 s, 55 °C for 30 s and 72 °C for 30 s, and a final extension step of 72 °C for 5 min. Samples were indexed using the Nextera® XT Index Kit v2 (Illumina, USA) and purified with MagSi-NGSprep Plus Beads (ratio 0.6 beads: 1 sample). The quality of the samples was assessed with a 5200 Fragment Analyzer System (Agilent Technologies, Santa Clara, USA). Each library was diluted to 4 nM and sequenced with the MiSeq Reagent kit v3 (600 cycles) and the Illumina MiSeq (Illumina, San Diego, CA, USA) device for paired-end sequencing (2 x 300).

Raw sequences were processed with fastp [8] v0.23.4 and default settings to remove sequencing adapters. All downstream processing, statistical analyses and data visualizations were performed within R v4.3.1. Briefly, sequences were processed with DADA2 [9] v1.28.031 until the obtention of amplicon sequencing variants (ASVs). The following parameters were selected along this process: forward and reverse reads were trimmed to 255 and 190 bp, respectively, where quality scores dropped below 28. Additionally, 20 and 16 nucleotides were trimmed from the start of the forward and reverse reads, respectively, to remove primer sequences. The resulting ASVs were taxonomically classified using a trained classifier from the SILVA v138.2 database at the 99% sequence identity using the ‘assignTaxonomy’ and ‘assignSpecies’ from the DADA2 [10] pipeline based on the RDP Naïve Bayes algorithm [11].

The output of the taxonomic assignation and the phylogenetic tree were exported into a phyloseq object with the phyloseq [12] v1.46.0 package. 28 ASVs from the negative controls were classified as contaminants when establishing a prevalence threshold of 0.1 and were subsequently removed from the samples using the decontam [13] v1.12 package. Sequences assigned to mitochondria, chloroplast, eukaryotes, NAs at the Kingdom level and singletons were also filtered out of the dataset using the ‘subset_taxa’ function of phyloseq. To assess whether a complete coverage of the bacterial communities was achieved with the selected sequencing depth, rarefaction curves were plotted using the ASVs table and the ‘rarecurve’ function of the vegan[14] v2.6-4 package.

Samples were rarefied to a depth of 5,000 sequences per sample prior to the alpha diversity assessment, which was the sequencing depth of the lowest-sequenced samples. Moreover, all samples reached a plateau in terms of ASVs at the selected sequencing depth, which demonstrates that this depth was enough to capture a good representation of bacterial diversity. Alpha diversity was characterized using Hill number; q = 0 (observed ASVs), q = 1 (exponential of Shannon index) and q = 2 (inverse Simpson index). The former was calculated using the ‘estimate_richness’ function of *phyloseq*, and q = 1 and q = 2 were calculated with the ‘diversity’ function of the vegan package together with subsequent calculations. Linear models were constructed for each Hill number using the ‘lm’ function of the stats v4.3.1 package, and differences in each Hill number were evaluated using Two-Way ANOVAs using the ‘anova’ function of the same package. Normality of residuals and homogeneity of variance were verified inspecting the Q-Q plots and the residuals-vs-fitted plots.

For the β-diversity analyses, sampling depth differences were corrected with the cumulative sum scaling (CSS) method using the ‘normalize’ function of the microbiomeMarker [15] v1.9.0 package. To assess differences in bacterial community composition across samples, dissimilarity was calculated with the Bray-Curtis distance through the ‘distance’ function of phyloseq and principal coordinates analyses (PCoA) were performed using the ‘ordinate’ function. Results were plotted with the ‘plot_ordination’ function. PERMANOVA tests (999 permutations) were carried out using the ‘adonis2’ function of the vegan package to further evaluate the effects of the study variables on bacterial community composition.

Differential abundance analyses at the ASV level were performed using DESeq2 [16] v1.40.2 and the non-normalized phyloseq object, applying the Wald test for significance and local fitting of dispersion estimates. Comparisons were made between samples from the sites under different land use types. We established a log₂ fold change threshold of ±2.5 and a Benjamini-Hochberg adjusted *P*-value threshold of 0.01. Results were plotted using ggplot2 [17] v3.5.1.

**Long-read metagenomic sequencing, processing and analyses**

Long-read metagenomic sequencing was carried out for taxonomic and functional potential profiling. Briefly, we deployed the Rapid Sequencing DNA V14 barcoding kit (SQK-RBK114.24) (Oxford Nanopore Technologies Ltd., Oxford, UK) for library preparation using 5 ng of each sample as input and the R10.4.1 PromethION Flow Cells (Oxford Nanopore Technologies Ltd., Oxford, UK) to sequence the extracted larval gut DNA. To enrich microbial DNA, adaptive sampling was performed to deplete host sequences. Reads mapping to the host reference genome (*Ephemera danica*; GCA_000507165.2, Edan_2.0) were selectively rejected in real-time.

Base-calling was performed with Dorado v0.7.2 (Oxford Nanopore Technologies Ltd., Oxford, UK) (r10.4.1_e8.2_400bps_hac@v5.0.0) in the high accuracy mode (hac) with the ‘basecaller’ function, adapters were removed and reads were demultiplexed based on the barcodes. Raw reads were quality filtered using chopper v0.2.1 with default settings using a minimum Phred quality score of 10, as implemented in previous studies [18,19]. The resulting reads were mapped against the previously used *E. danica* assembly (GCA_000507165.2, Edan_2.0) using Minimap2 [20] v2.28 and the ‘map-ont’ option for noisy long reads. The unmapped reads were sequentially split using SAMtools [21] v1.9. The same approach was carried out to remove the sequences present in the negative controls.

The filtered reads were co-assembled by combining the samples from the same site and sampling time using metaMDBG [22] v1.1, which has shown to outperform other long-read assemblers [22]. The resulting contigs were processed within the SqueezeMeta [23] v1.7.0 pipeline for functional annotation. Briefly, open reading frames (ORFs) were predicted using Prodigal [24] v2.6.3, and annotated using DIAMOND [25] v2.0.15 against the GenBank non-redundant (nr) and KEGG databases with a minimum e-value threshold of 10^-5^. Additional ORFs were detected using DIAMOND BlastX [25]. The taxonomic assignment of predicted genes was performed with the Lowest Common Ancestor (LCA) algorithm [26] based on DIAMOND alignment results. The processed reads were mapped against the contigs using Minimap2 to estimate coverage and gene abundance.

The same pipeline was deployed to retrieve metagenome assembled genomes (MAGs), using its binning module with MetaBAT [27] v2.12.1, MaxBin [28] v2.2.6, and CONCOCT [29] v1.0.0. A non-redundant set of bins was retrieved using DASTool [30] v1.1.7, then polished with MAGpurify (<https://github.com/snayfach/MAGpurify>) v2.1.2, and evaluated for completeness and contamination using CheckM2 [31] v1.1.0. Finally, MAGs with >40% completeness and <10% contamination were taxonomically classified using GTDB-Tk [32] v2.1.0 and the Genome Taxonomy Database (GTDB), and functionally annotated using DRAM [33] v1.4.0. The coverage of each MAG across samples was calculated using CoverM [34] v0.7.0.

The ORF tables generated by SqueezeMeta were imported into R and filtered using KEGG IDs corresponding to genes linked to degradation and host-microbe interactions (listed in Table S3) and the taxa names obtained in the differential abundance analyses from the 16S rRNA amplicon dataset. For each gene of interest, mapped bases were calculated as (gene coverage × gene length), divided by the total bases per sample, and scaled to counts per million (1 × 10^6^). The MAGs analyses results were brought together using the pheatmap [35] package.

**Supplementary figures**

**Figure S1**: Gut dissection of one specimen of mayfly larvae (Ephemera danica).

**Figure S2: Alpha diversity of larval gut bacterial communities across sites and sampling times**. Sites include extensive grassland (EO), intensive farming (IO) and forest (FO). Diversity was quantified with Hill numbers (q = 0, 1 and 2) using the rarefied dataset (5,407 reads). Each point represents a single sample, and horizontal bars indicate group means. Statistical differences were assessed using Two-way ANOVAs, with effect size as η2, taking into account the site and sampling time (ST) variables, and their interaction.

**Figure S4: Within-group bacterial community heterogeneity based on the 16S rRNA amplicon dataset.** Multivariate dispersion (distance-to-centroid) calculated from Bray-Curtis distances. Results show variation in community stability between the extensive grassland (EO), intensive farming (IO) and forest sites (FO) across the summer (T1) and autumn (T2) sampling times. Differences were assessed with the Kruskal-Wallis test.

**Figure S3:** Monthly accumulated rainfall (mm) in 2023, displayed as barplots, with a line indicating average daily rainfall (mm). The red arrows indicate when the sampling events took place.

**Figure S5: Abundance of functional groups across sites and sampling times.** Average and standard deviation of the abundance in copies per million bases (CPMB) of degradative and microbial-host interactions genes identified across the extensive grassland (EO), intensive farming (IO) and forest (FO) sites along the Otterbach stream, at the summer (T1) and autumn (T2) sampling time.

**Figure S6: Genomic context of the detected genes of interest within the retrieved MAGs and their contigs**. Colour according to the gene type: gene of interest and neighbour gene. Above the taxonomic classification of the MAGs.

**Supplementary tables**

**Table S1:** Overview of 16S rRNA amplicon sequencing reads retention across DADA2 processing steps, including filtering, denoising, and chimera removal, and after the filtering of contaminants with decontam.

| Sample ID | DADA2 Workflow | | | | | | Decontam | % | Sample type |
| --- | --- | --- | --- | --- | --- | --- | --- | --- | --- |
|  | Input | Filtered | DenoisedF | DenoisedR | Merged | Nonchim | After_filt |  |  |
| RM-105 | 221 | 134 | 100 | 106 | 92 | 92 | 0 | 0 | extraction_neg |
| RM-106 | 510 | 354 | 309 | 295 | 273 | 273 | 0 | 0 | pcr_neg |
| RM-11 | 18799 | 13418 | 11572 | 12054 | 7423 | 7130 | 6958 | 97.59 | sample |
| RM-12 | 26532 | 18602 | 15947 | 16867 | 9832 | 9674 | 9385 | 97.01 | sample |
| RM-13 | 46251 | 33223 | 30532 | 31136 | 23989 | 23026 | 22408 | 97.32 | sample |
| RM-14 | 32287 | 22719 | 20593 | 21120 | 14299 | 13416 | 12683 | 94.54 | sample |
| RM-142 | 46321 | 33461 | 32382 | 32673 | 30417 | 25046 | 24875 | 99.32 | sample |
| RM-143 | 72102 | 50551 | 49060 | 49414 | 46036 | 38895 | 38575 | 99.18 | sample |
| RM-144 | 55487 | 39507 | 38446 | 38813 | 36780 | 29876 | 29744 | 99.56 | sample |
| RM-145 | 63309 | 41455 | 39157 | 39902 | 33712 | 28346 | 27510 | 97.05 | sample |
| RM-146 | 57670 | 41418 | 40170 | 40427 | 37866 | 32128 | 32026 | 99.68 | sample |
| RM-147 | 74056 | 52331 | 51153 | 51586 | 48344 | 40052 | 40032 | 99.95 | sample |
| RM-148 | 77059 | 56623 | 55401 | 55959 | 52998 | 42925 | 42799 | 99.71 | sample |
| RM-149 | 36480 | 26349 | 25703 | 25765 | 24243 | 21164 | 21036 | 99.40 | sample |
| RM-15 | 29250 | 20697 | 17740 | 18514 | 10140 | 9928 | 9605 | 96.75 | sample |
| RM-150 | 60296 | 43204 | 42732 | 42525 | 40684 | 37368 | 35302 | 94.47 | sample |
| RM-151 | 35675 | 25495 | 24652 | 24947 | 23169 | 19889 | 19836 | 99.73 | sample |
| RM-152 | 33491 | 24255 | 23227 | 23399 | 20891 | 17624 | 17517 | 99.39 | sample |
| RM-153 | 26298 | 18702 | 17542 | 17863 | 15379 | 13645 | 13585 | 99.56 | sample |
| RM-154 | 37282 | 26389 | 24842 | 25161 | 22332 | 19324 | 18975 | 98.19 | sample |
| RM-155 | 36505 | 25057 | 24012 | 24354 | 22732 | 19174 | 18992 | 99.05 | sample |
| RM-156 | 54161 | 38797 | 38013 | 38244 | 36241 | 30069 | 29977 | 99.69 | sample |
| RM-16 | 44679 | 31973 | 30146 | 30803 | 26825 | 22797 | 22657 | 99.39 | sample |
| RM-17 | 44047 | 32018 | 28639 | 29637 | 19212 | 18390 | 17564 | 95.51 | sample |
| RM-18 | 43554 | 31490 | 28738 | 29694 | 22130 | 20190 | 19331 | 95.75 | sample |
| RM-19 | 28220 | 20793 | 18869 | 19195 | 14224 | 13107 | 12861 | 98.12 | sample |
| RM-20 | 44850 | 33053 | 31365 | 31839 | 28107 | 24511 | 24431 | 99.67 | sample |
| RM-21 | 40999 | 29952 | 26788 | 27652 | 18767 | 17549 | 16938 | 96.52 | sample |
| RM-214 | 50373 | 35720 | 34510 | 34416 | 28947 | 26924 | 0 | 0 | extraction_neg |
| RM-215 | 1688 | 1178 | 1087 | 1112 | 953 | 926 | 0 | 0 | extraction_neg |
| RM-216 | 2306 | 1612 | 1463 | 1438 | 1204 | 1184 | 0 | 0 | extraction_neg |
| RM-217 | 255 | 178 | 167 | 164 | 163 | 163 | 0 | 0 | pcr_neg |
| RM-22 | 18045 | 13026 | 10766 | 11364 | 6036 | 5937 | 5047 | 85.01 | sample |
| RM-23 | 40740 | 27644 | 25073 | 25959 | 20164 | 18391 | 18046 | 98.12 | sample |
| RM-236 | 3206 | 2196 | 1905 | 1881 | 1441 | 1344 | 0 | 0 | extraction_neg |
| RM-237 | 390 | 241 | 184 | 156 | 154 | 154 | 0 | 0 | pcr_neg |
| RM-238 | 7114 | 5067 | 4687 | 4753 | 4166 | 3976 | 0 | 0 | pbs_neg |
| RM-24 | 107544 | 78796 | 75561 | 76590 | 66956 | 57711 | 57427 | 99.51 | sample |
| RM-25 | 26580 | 18939 | 17321 | 17752 | 14839 | 13444 | 13395 | 99.64 | sample |
| RM-93 | 661 | 466 | 434 | 439 | 419 | 419 | 0 | 0 | extraction_neg |
| RM-94 | 2710 | 1952 | 1748 | 1665 | 1404 | 1371 | 0 | 0 | extraction_neg |
| RM-95 | 2114 | 1526 | 1381 | 1335 | 1048 | 1034 | 0 | 0 | extraction_neg |
| RM-96 | 510 | 362 | 331 | 319 | 286 | 286 | 0 | 0 | pcr_neg |

**Table S2:** Overview of the long-read metagenomic sequencing raw files before processing, including the initial number of sequences (num_seqs), the sum of the total length (sum_len), the minimum read length (min_len), the average length (avg_len) and the maximum read length (max_len).

| File | Num_seqs | Sum_len |  | Min Len | Avg_Len | Max Len |
| --- | --- | --- | --- | --- | --- | --- |
| SQK-RBK114-24_barcode01.fastq | 1,639,527 | 473,611,002 |  | 61 | 288.9 | 550,830 |
| SQK-RBK114-24_barcode02.fastq | 1,127,347 | 357,393,153 |  | 61 | 317 | 678,692 |
| SQK-RBK114-24_barcode03.fastq | 1,874,628 | 616,044,011 |  | 33 | 328.6 | 560,446 |
| SQK-RBK114-24_barcode04.fastq | 4,439,769 | 3,013,624,947 |  | 57 | 678.8 | 967,051 |
| SQK-RBK114-24_barcode05.fastq | 1,525,976 | 562,644,447 |  | 62 | 368.7 | 707,598 |
| SQK-RBK114-24_barcode06.fastq | 1,493,774 | 412,502,657 |  | 62 | 276.1 | 887,023 |
| SQK-RBK114-24_barcode07.fastq | 1,619,108 | 421,544,653 |  | 57 | 260.4 | 467,414 |
| SQK-RBK114-24_barcode08.fastq | 2,457,345 | 2,107,992,535 |  | 27 | 857.8 | 831,644 |
| SQK-RBK114-24_barcode09.fastq | 115,799 | 20,094,800 |  | 60 | 173.5 | 218,928 |
| SQK-RBK114-24_barcode10.fastq | 726,142 | 248,935,863 |  | 61 | 342.8 | 701,529 |
| SQK-RBK114-24_barcode11.fastq | 934,419 | 219,506,214 |  | 59 | 234.9 | 592,286 |
| SQK-RBK114-24_barcode12.fastq | 660,666 | 252,954,008 |  | 30 | 382.9 | 574,643 |
| SQK-RBK114-24_barcode13.fastq | 2,144,866 | 519,000,874 |  | 31 | 242 | 454,018 |
| SQK-RBK114-24_barcode14.fastq | 1,801,609 | 501,257,394 |  | 48 | 278.2 | 627,155 |
| SQK-RBK114-24_barcode15.fastq | 569,971 | 99,605,856 |  | 28 | 174.8 | 974,317 |
| SQK-RBK114-24_barcode16.fastq | 1,376,507 | 474,398,088 |  | 59 | 344.6 | 662,363 |
| SQK-RBK114-24_barcode17.fastq | 12,074 | 7,112,963 |  | 74 | 589.1 | 38,652 |
| SQK-RBK114-24_barcode18.fastq | 12,261 | 9,668,271 |  | 80 | 788.5 | 59,111 |
| SQK-RBK114-24_barcode19.fastq | 7,658 | 1,843,331 |  | 63 | 240.7 | 4,397 |
| SQK-RBK114-24_barcode20.fastq | 172 | 90,897 |  | 49 | 528.5 | 5,490 |
| SQK-RBK114-24_barcode21.fastq | 306 | 199,334 |  | 81 | 651.4 | 39,235 |
| SQK-RBK114-24_barcode22.fastq | 20,973 | 5,298,127 |  | 73 | 252.6 | 166,180 |
| SQK-RBK114-24_barcode23.fastq | 17,761 | 5,092,912 |  | 71 | 286.7 | 127,457 |
| SQK-RBK114-24_barcode24.fastq | 18,695 | 7,822,339 |  | 67 | 418.4 | 104,445 |
| SQK-RBK114-24_barcode01.fastq | 3,295,482 | 887,489,869 |  | 63 | 269.3 | 679,648 |
| SQK-RBK114-24_barcode02.fastq | 3,172,567 | 1,341,945,021 |  | 59 | 423 | 480,115 |
| SQK-RBK114-24_barcode03.fastq | 1,043,267 | 256,857,047 |  | 58 | 246.2 | 617,790 |
| SQK-RBK114-24_barcode04.fastq | 853,964 | 255,105,325 |  | 60 | 298.7 | 585,118 |
| SQK-RBK114-24_barcode05.fastq | 1,192,386 | 478,652,619 |  | 70 | 401.4 | 727,967 |
| SQK-RBK114-24_barcode06.fastq | 1,106,363 | 291,654,398 |  | 56 | 263.6 | 630,935 |
| SQK-RBK114-24_barcode07.fastq | 1,140,550 | 266,829,635 |  | 59 | 233.9 | 451,440 |
| SQK-RBK114-24_barcode08.fastq | 14,630 | 6,578,511 |  | 70 | 449.7 | 702,151 |
| SQK-RBK114-24_barcode09.fastq | 841 | 188,333 |  | 64 | 223.9 | 37,761 |
| SQK-RBK114-24_barcode10.fastq | 121,608 | 36,838,448 |  | 62 | 302.9 | 510,656 |
| SQK-RBK114-24_barcode11.fastq | 1,051,600 | 256,409,844 |  | 67 | 243.8 | 599,186 |
| SQK-RBK114-24_barcode12.fastq | 832,163 | 294,277,427 |  | 66 | 353.6 | 611,468 |
| SQK-RBK114-24_barcode13.fastq | 331,206 | 63,522,217 |  | 56 | 191.8 | 248,461 |
| SQK-RBK114-24_barcode14.fastq | 1,976,690 | 628,582,552 |  | 46 | 318 | 834,265 |
| SQK-RBK114-24_barcode15.fastq | 1,157,009 | 261,314,750 |  | 38 | 225.9 | 374,412 |
| SQK-RBK114-24_barcode16.fastq | 402,073 | 83,761,215 |  | 59 | 208.3 | 695,697 |
| SQK-RBK114-24_barcode17.fastq | 1,624,882 | 451,828,494 |  | 60 | 278.1 | 1,193,814 |
| SQK-RBK114-24_barcode18.fastq | 1,620,994 | 564,898,760 |  | 65 | 348.5 | 418,877 |
| SQK-RBK114-24_barcode19.fastq | 1,689,433 | 432,249,966 |  | 61 | 255.9 | 409,616 |
| SQK-RBK114-24_barcode20.fastq | 134,398 | 28,001,660 |  | 63 | 208.3 | 291,006 |
| SQK-RBK114-24_barcode21.fastq | 109 | 70,261 |  | 75 | 644.6 | 41,330 |
| SQK-RBK114-24_barcode22.fastq | 169 | 40,132 |  | 68 | 237.5 | 1,709 |
| SQK-RBK114-24_barcode23.fastq | 99 | 21,336 |  | 81 | 215.5 | 650 |
| SQK-RBK114-24_barcode24.fastq | 98 | 25,596 |  | 76 | 261.2 | 3,185 |

**Table S3:** Selection of genes that we used to subset the taxonomic annotation results and could somehow be related to adjacent land use, along with their functional category, broader functional category and KEGG ID.

| KEGG.ID | Gene | Function | Subgroup | Group |
| --- | --- | --- | --- | --- |
| K20844 | abf1 | hemicellulose_degradation | complex_carbon_xenobiotic_degradation | complex_carbon_xenobiotic_degradation |
| K01209 | abfA | hemicellulose_degradation | complex_carbon_xenobiotic_degradation | complex_carbon_xenobiotic_degradation |
| K05394 | atzA | atrazine_degradation | xenobiotics_degradation | complex_carbon_xenobiotic_degradation |
| K03382 | atzB | atrazine_degradation | xenobiotics_degradation | complex_carbon_xenobiotic_degradation |
| K03383 | atzD | atrazine_degradation | xenobiotics_degradation | complex_carbon_xenobiotic_degradation |
| K20542 | bcsZ | cellulose_degradation | complex_carbon_xenobiotic_degradation | complex_carbon_xenobiotic_degradation |
| K05549 | benA | aromatic_compounds_degradation | complex_carbon_xenobiotic_degradation | complex_carbon_xenobiotic_degradation |
| K05550 | benB | aromatic_compounds_degradation | complex_carbon_xenobiotic_degradation | complex_carbon_xenobiotic_degradation |
| K05784 | benC | aromatic_compounds_degradation | complex_carbon_xenobiotic_degradation | complex_carbon_xenobiotic_degradation |
| K05783 | benD | aromatic_compounds_degradation | complex_carbon_xenobiotic_degradation | complex_carbon_xenobiotic_degradation |
| K01223 | bglA | hemicellulose_degradation | complex_carbon_xenobiotic_degradation | complex_carbon_xenobiotic_degradation |
| K05349 | bglX | hemicellulose_degradation | complex_carbon_xenobiotic_degradation | complex_carbon_xenobiotic_degradation |
| K15511 | boxA | aromatic_compounds_degradation | complex_carbon_xenobiotic_degradation | complex_carbon_xenobiotic_degradation |
| K15512 | boxB | aromatic_compounds_degradation | complex_carbon_xenobiotic_degradation | complex_carbon_xenobiotic_degradation |
| K15513 | boxC | aromatic_compounds_degradation | complex_carbon_xenobiotic_degradation | complex_carbon_xenobiotic_degradation |
| K03381 | catA | aromatic_compounds_degradation | complex_carbon_xenobiotic_degradation | complex_carbon_xenobiotic_degradation |
| K01856 | catB | aromatic_compounds_degradation | complex_carbon_xenobiotic_degradation | complex_carbon_xenobiotic_degradation |
| K07104 | catE | aromatic_compounds_degradation | complex_carbon_xenobiotic_degradation | complex_carbon_xenobiotic_degradation |
| K08095 | CUT1_2_3 | pectine_cutine_degradation | complex_carbon_xenobiotic_degradation | complex_carbon_xenobiotic_degradation |
| K16249 | dmpK | aromatic_compounds_degradation | complex_carbon_xenobiotic_degradation | complex_carbon_xenobiotic_degradation |
| K16243 | dmpL | aromatic_compounds_degradation | complex_carbon_xenobiotic_degradation | complex_carbon_xenobiotic_degradation |
| K16244 | dmpM | aromatic_compounds_degradation | complex_carbon_xenobiotic_degradation | complex_carbon_xenobiotic_degradation |
| K16242 | dmpN | aromatic_compounds_degradation | complex_carbon_xenobiotic_degradation | complex_carbon_xenobiotic_degradation |
| K16245 | dmpO | aromatic_compounds_degradation | complex_carbon_xenobiotic_degradation | complex_carbon_xenobiotic_degradation |
| K16246 | dmpP | aromatic_compounds_degradation | complex_carbon_xenobiotic_degradation | complex_carbon_xenobiotic_degradation |
| K01179 | EG | cellulose_degradation | complex_carbon_xenobiotic_degradation | complex_carbon_xenobiotic_degradation |
| K04099 | galA | hemicellulose_degradation | complex_carbon_xenobiotic_degradation | complex_carbon_xenobiotic_degradation |
| K00104 | gox | glyphosate_degradation: | xenobiotics_degradation | complex_carbon_xenobiotic_degradation |
| K00799 | GST | aromatic_compounds_degradation | complex_carbon_xenobiotic_degradation | complex_carbon_xenobiotic_degradation |
| K04100 | ligA | aromatic_compounds_degradation | complex_carbon_xenobiotic_degradation | complex_carbon_xenobiotic_degradation |
| K10219 | ligC | aromatic_compounds_degradation | complex_carbon_xenobiotic_degradation | complex_carbon_xenobiotic_degradation |
| K01857 | pcaB | aromatic_compounds_degradation | complex_carbon_xenobiotic_degradation | complex_carbon_xenobiotic_degradation |
| K01607 | pcaC | aromatic_compounds_degradation | complex_carbon_xenobiotic_degradation | complex_carbon_xenobiotic_degradation |
| K00448 | pcaG | aromatic_compounds_degradation | complex_carbon_xenobiotic_degradation | complex_carbon_xenobiotic_degradation |
| K01966 | pccA | aromatic_compounds_degradation | complex_carbon_xenobiotic_degradation | complex_carbon_xenobiotic_degradation |
| K01184 | pehA | pectine_cutine_degradation | complex_carbon_xenobiotic_degradation | complex_carbon_xenobiotic_degradation |
| K01728 | pel | pectine_cutine_degradation | complex_carbon_xenobiotic_degradation | complex_carbon_xenobiotic_degradation |
| K19551 | pelC | pectine_cutine_degradation | complex_carbon_xenobiotic_degradation | complex_carbon_xenobiotic_degradation |
| K06163 | phnJ | glyphosate_degradation: | xenobiotics_degradation | complex_carbon_xenobiotic_degradation |
| K22539 | PLY | pectine_cutine_degradation | complex_carbon_xenobiotic_degradation | complex_carbon_xenobiotic_degradation |
| K10759 | tanA | aromatic_compounds_degradation | complex_carbon_xenobiotic_degradation | complex_carbon_xenobiotic_degradation |
| K27357 | tfH | pectine_cutine_degradation | complex_carbon_xenobiotic_degradation | complex_carbon_xenobiotic_degradation |
| K01181 | xyn | hemicellulose_degradation | complex_carbon_xenobiotic_degradation | complex_carbon_xenobiotic_degradation |
| K01198 | xynB | hemicellulose_degradation | complex_carbon_xenobiotic_degradation | complex_carbon_xenobiotic_degradation |
| K15921 | xynD | hemicellulose_degradation | complex_carbon_xenobiotic_degradation | complex_carbon_xenobiotic_degradation |
| K03585 | mexA | efflux_pumps | efflux_pumps | efflux_pumps |
| K18138 | mexB | efflux_pumps | efflux_pumps | efflux_pumps |
| K18139 | oprM | efflux_pumps | efflux_pumps | efflux_pumps |
| K12340 | tolC | efflux_pumps | efflux_pumps | efflux_pumps |
| K00925 | ackA | acetate kinase | acetate_production | SCFA_production |
| K15023 | acsE | CO dehydrogenase/acetyl-CoA synthase complex | acetate_production | SCFA_production |
| K23259 | adh | 1-propanol dehydrogenase | propionate_production | SCFA_production |
| K01035 | atoA | acetate CoA/acetoacetate CoA-transferase beta subunit | butyrate_production | SCFA_production |
| K01034 | atoD | acetate CoA/acetoacetate CoA-transferase alpha subunit | butyrate_production | SCFA_production |
| K00929 | buk | butyrate kinase | butyrate_production | SCFA_production |
| K00192 | cdhA | CO dehydrogenase/acetyl-CoA synthase | acetate_production | SCFA_production |
| K00195 | cdhB | CO dehydrogenase/acetyl-CoA synthase | acetate_production | SCFA_production |
| K00193 | cdhC | CO dehydrogenase/acetyl-CoA synthase | acetate_production | SCFA_production |
| K00194 | cdhD | CO dehydrogenase/acetyl-CoA synthase | acetate_production | SCFA_production |
| K00197 | cdhE | CO dehydrogenase/acetyl-CoA synthase | acetate_production | SCFA_production |
| K01026 | pct | propionate CoA-transferase | propionate_production | SCFA_production |
| K01699 | pduC | propanediol dehydratase | propionate_production | SCFA_production |
| K13919 | pduD | propanediol dehydratase | propionate_production | SCFA_production |
| K13920 | pduE | propanediol dehydratase | propionate_production | SCFA_production |
| K13922 | pduP | propionaldehyde dehydrogenase | propionate_production | SCFA_production |
| K19697 | pduW | propionate kinase | propionate_production | SCFA_production |
| K03737 | por | pyruvate:ferredoxin oxidoreductase | propionate_production | SCFA_production |
| K00625 | pta | phosphate acetyltransferase | acetate_production | SCFA_production |
| K13788 | pta | phosphate acetyltransferase | acetate_production | SCFA_production |
| K00634 | ptb | phosphate butyryltransferase | butyrate_production | SCFA_production |
| K10804 | tesA | acyl-CoA thioesterase | acetate_kinase | SCFA_production |
| K10805 | tesB | acyl-CoA thioesterase | acetate_kinase | SCFA_production |
| K10806 | yciA | acyl-CoA thioesterase | acetate_kinase | SCFA_production |
| K19709 | ydiF | acetate CoA-transferase | butyrate_production | SCFA_production |
| K14171 | ahpC | alkyl hydroperoxide reductase | stress_response | stress_response |
| K00383 | gor | glutathione reductase | stress_response | stress_response |
| K03781 | katE | catalase | stress_response | stress_response |
| K03782 | katG | catalase | stress_response | stress_response |
| K04761 | oxyR | oxidative stress transcriptional regulator | stress_response | stress_response |
| K04564 | sodA | superoxide dismutase | stress_response | stress_response |
| K13639 | soxR | redox-sensitive regulators | stress_response | stress_response |
| K13631 | soxS | redox-sensitive regulators | stress_response | stress_response |

**Table S4:** Kruskal-Wallis comparisons of the abundances of the selected gene groups between sites at each sampling time (ST).

| ST | Gene group | *P-*adj value | signif |
| --- | --- | --- | --- |
| T1 | SCFA production | 0.0144 | ** |
| T1 | Complex carbon & xenobiotic degradation | 0.00492 | *** |
| T1 | Efflux pumps | 0.273 | ns |
| T1 | Stress response | 0.01002 | * |
| T2 | SCFA production | 0.00492 | *** |
| T2 | Complex carbon xenobiotic degradation | 0.273 | ns |
| T2 | Efflux pumps | 0.0083733 | ** |
| T2 | Stress response | 0.0144 | * |

**Table S5:** Dunn post-hoc comparing the abundances of the selected gene groups between sites at each sampling time (ST) for the ones that showed significant differences in the Kruskal-Wallis.

| ST | Gene group | group1 | group2 | *P*-adj value | signif |
| --- | --- | --- | --- | --- | --- |
| T1 | SCFA production | EO | FO | 0.970302 | ns |
| T1 | SCFA production | EO | IO | 0.008637 | ** |
| T1 | SCFA production | FO | IO | 0.008637 | ** |
| T1 | Complex carbon xenobiotic degradation | EO | FO | 0.068364 | ns |
| T1 | Complex carbon xenobiotic degradation | EO | IO | 0.000523 | *** |
| T1 | Complex carbon xenobiotic degradation | FO | IO | 0.05071 | ns |
| T1 | Stress response | EO | FO | 0.10055 | ns |
| T1 | Stress response | EO | IO | 0.004462 | ** |
| T1 | Stress response | FO | IO | 0.10055 | ns |
| T2 | SCFA production | EO | FO | 0.479038 | ns |
| T2 | SCFA production | EO | IO | 0.023678 | * |
| T2 | SCFA production | FO | IO | 0.000857 | *** |
| T2 | Efflux pumps | EO | FO | 0.006565 | ** |
| T2 | Efflux pumps | EO | IO | 0.654714 | ns |
| T2 | Efflux pumps | FO | IO | 0.006565 | ** |
| T2 | Stress response | EO | FO | 0.88664 | ns |
| T2 | Stress response | EO | IO | 0.007855 | ** |
| T2 | Stress response | FO | IO | 0.007855 | ** |

**References**

1.	Martínez-Cuesta R, Hoess R, Floßmann S, Geist J, Dannenmann M, Schloter M, *et al.* Agricultural land use shapes short and long‑term bacterial diversity, community structure, and assembly in biofilms of adjacent streams. *Environ Microbiome* 2026; https://doi.org/10.1186/s40793-025-00837-9.

2.	Angstmann H, Pfeiffer S, Kublik S, Ehrhardt B, Uliczka K, Rabe KF, *et al.* The microbial composition of larval airways from Drosophila melanogaster differ between specimens from laboratory and natural habitats. *Environ Microbiome* 2023;**18**:55. https://doi.org/10.1186/s40793-023-00506-9.

3.	Ferguson L V., Dhakal P, Lebenzon JE, Heinrichs DE, Bucking C, Sinclair BJ. Seasonal shifts in the insect gut microbiome are concurrent with changes in cold tolerance and immunity. *Funct Ecol* 2018;**32**:2357–68. https://doi.org/10.1111/1365-2435.13153.

4.	Vacheron J, Péchy-Tarr M, Brochet S, Heiman CM, Stojiljkovic M, Maurhofer M, *et al.* T6SS contributes to gut microbiome invasion and killing of an herbivorous pest insect by plant-beneficial *Pseudomonas protegens*. *ISME J* 2019;**13**:1318–29. https://doi.org/10.1038/s41396-019-0353-8.

5.	Martínez-Cuesta R, Holmer A, Buegger F, Dannenmann M, Schloter M, Schulz S. Land use drives prokaryotic community composition of directly adjacent grasslands. *Biol Fertil Soils* 2024;**61**:71–84. https://doi.org/10.1007/s00374-024-01871-4.

6.	Klindworth A, Pruesse E, Schweer T, Peplies J, Quast C, Horn M, *et al.* Evaluation of general 16S ribosomal RNA gene PCR primers for classical and next-generation sequencing-based diversity studies. *Nucleic Acids Res* 2013;**41**:e1–e1. https://doi.org/10.1093/nar/gks808.

7.	Streb L-M, Cholewińska P, Gschwendtner S, Geist J, Rath S, Schloter M. Age matters: exploring differential effects of antimicrobial treatment on gut microbiota of adult and juvenile brown trout (*Salmo trutta*). *Anim Microbiome* 2025;**7**:28. https://doi.org/10.1186/s42523-025-00391-2.

8.	Chen S, Zhou Y, Chen Y, Gu J. fastp: an ultra-fast all-in-one FASTQ preprocessor. *Bioinformatics* 2018;**34**:i884–90. https://doi.org/10.1093/bioinformatics/bty560.

9.	Callahan BJ, McMurdie PJ, Rosen MJ, Han AW, Johnson AJA, Holmes SP. DADA2: High-resolution sample inference from Illumina amplicon data. *Nat Methods* 2016;**13**:581–83. https://doi.org/10.1038/nmeth.3869.

10.	Callahan BJ, McMurdie PJ, Rosen MJ, Han AW, Johnson AJA, Holmes SP. DADA2: High-resolution sample inference from Illumina amplicon data. *Nat Methods* 2016;**13**:581–83. https://doi.org/https://doi.org/10.1038/nmeth.3869.

11.	Wang Q, Garrity GM, Tiedje JM, Cole JR. Naïve Bayesian Classifier for Rapid Assignment of rRNA Sequences into the New Bacterial Taxonomy. *Appl Environ Microbiol* 2007;**73**:5261–67. https://doi.org/10.1128/AEM.00062-07.

12.	McMurdie PJ, Holmes S. phyloseq: An R Package for Reproducible Interactive Analysis and Graphics of Microbiome Census Data. *PLoS One* 2013;**8**:e61217. https://doi.org/10.1371/journal.pone.0061217.

13.	Davis NM, Proctor DM, Holmes SP, Relman DA, Callahan BJ. Simple statistical identification and removal of contaminant sequences in marker-gene and metagenomics data. *Microbiome* 2018;**6**:226. https://doi.org/10.1186/s40168-018-0605-2.

14.	Oksanen J, Kindt R, Legendre P, O’Hara B, Simpson G, Solymos P, *et al.* The vegan package.

15.	Cao Y, Dong Q, Wang D, Zhang P, Liu Y, Niu C. microbiomeMarker: an R/Bioconductor package for microbiome marker identification and visualization. *Bioinformatics* 2022;**38**:4027–29. https://doi.org/10.1093/bioinformatics/btac438.

16.	Love MI, Huber W, Anders S. Moderated estimation of fold change and dispersion for RNA-seq data with DESeq2. *Genome Biol* 2014;**15**:550. https://doi.org/10.1186/s13059-014-0550-8.

17.	Wickham H. ggplot2. *WIREs Computational Statistics* 2011;**3**:180–85. https://doi.org/10.1002/wics.147.

18.	Reska T, Pozdniakova S, Borràs S, Perlas A, Sauerborn E, Cañas L, *et al.* Air monitoring by nanopore sequencing. *ISME Communications* 2024;**4**. https://doi.org/10.1093/ismeco/ycae099.

19.	Sauerborn E, Corredor NC, Reska T, Perlas A, Vargas da Fonseca Atum S, Goldman N, *et al.* Detection of hidden antibiotic resistance through real-time genomics. *Nat Commun* 2024;**15**:5494. https://doi.org/10.1038/s41467-024-49851-4.

20.	Li H. Minimap2: pairwise alignment for nucleotide sequences. *Bioinformatics* 2018;**34**:3094–100. https://doi.org/10.1093/bioinformatics/bty191.

21.	Li H, Handsaker B, Wysoker A, Fennell T, Ruan J, Homer N, *et al.* The Sequence Alignment/Map format and SAMtools. *Bioinformatics* 2009;**25**:2078–79. https://doi.org/10.1093/bioinformatics/btp352.

22.	Benoit G, Raguideau S, James R, Phillippy AM, Chikhi R, Quince C. High-quality metagenome assembly from long accurate reads with metaMDBG. *Nat Biotechnol* 2024;**42**:1378–83. https://doi.org/10.1038/s41587-023-01983-6.

23.	Tamames J, Puente-Sánchez F. SqueezeMeta, A Highly Portable, Fully Automatic Metagenomic Analysis Pipeline. *Front Microbiol* 2019;**9**. https://doi.org/10.3389/fmicb.2018.03349.

24.	Hyatt D, Chen G-L, LoCascio PF, Land ML, Larimer FW, Hauser LJ. Prodigal: prokaryotic gene recognition and translation initiation site identification. *BMC Bioinformatics* 2010;**11**:119. https://doi.org/10.1186/1471-2105-11-119.

25.	Buchfink B, Reuter K, Drost H-G. Sensitive protein alignments at tree-of-life scale using DIAMOND. *Nat Methods* 2021;**18**:366–68. https://doi.org/10.1038/s41592-021-01101-x.

26.	Hanson NW, Konwar KM, Hallam SJ. LCA*: an entropy-based measure for taxonomic assignment within assembled metagenomes. *Bioinformatics* 2016;**32**:3535–42. https://doi.org/10.1093/bioinformatics/btw400.

27.	Kang DD, Li F, Kirton E, Thomas A, Egan R, An H, *et al.* MetaBAT 2: an adaptive binning algorithm for robust and efficient genome reconstruction from metagenome assemblies. *PeerJ* 2019;**7**:e7359. https://doi.org/10.7717/peerj.7359.

28.	Wu Y-W, Simmons BA, Singer SW. MaxBin 2.0: an automated binning algorithm to recover genomes from multiple metagenomic datasets. *Bioinformatics* 2016;**32**:605–07. https://doi.org/10.1093/bioinformatics/btv638.

29.	Alneberg J, Bjarnason BS, de Bruijn I, Schirmer M, Quick J, Ijaz UZ, *et al.* Binning metagenomic contigs by coverage and composition. *Nat Methods* 2014;**11**:1144–46. https://doi.org/10.1038/nmeth.3103.

30.	Sieber CMK, Probst AJ, Sharrar A, Thomas BC, Hess M, Tringe SG, *et al.* Recovery of genomes from metagenomes via a dereplication, aggregation and scoring strategy. *Nat Microbiol* 2018;**3**:836–43. https://doi.org/10.1038/s41564-018-0171-1.

31.	Chklovski A, Parks DH, Woodcroft BJ, Tyson GW. CheckM2: a rapid, scalable and accurate tool for assessing microbial genome quality using machine learning. *Nat Methods* 2023;**20**:1203–12. https://doi.org/10.1038/s41592-023-01940-w.

32.	Chaumeil P-A, Mussig AJ, Hugenholtz P, Parks DH. GTDB-Tk: a toolkit to classify genomes with the Genome Taxonomy Database. *Bioinformatics* 2020;**36**:1925–27. https://doi.org/10.1093/bioinformatics/btz848.

33.	Shaffer M, Borton MA, McGivern BB, Zayed AA, La Rosa SL, Solden LM, *et al.* DRAM for distilling microbial metabolism to automate the curation of microbiome function. *Nucleic Acids Res* 2020;**48**:8883–900. https://doi.org/10.1093/nar/gkaa621.

34.	Aroney STN, Newell RJP, Nissen JN, Camargo AP, Tyson GW, Woodcroft BJ. CoverM: read alignment statistics for metagenomics. *Bioinformatics* 2025;**41**. https://doi.org/10.1093/bioinformatics/btaf147.

35.	Gu Z, Eils R, Schlesner M. Complex heatmaps reveal patterns and correlations in multidimensional genomic data. *Bioinformatics* 2016;**32**:2847–49. https://doi.org/10.1093/bioinformatics/btw313.
